# Risk Selection, Risk Adjustment and Choice: Concepts and Lessons from the Americas

**DOI:** 10.3390/ijerph10115299

**Published:** 2013-10-25

**Authors:** Randall P. Ellis, Juan Gabriel Fernandez

**Affiliations:** 1 Departement of Economics, Boston University, 270 Bay State Road, Boston, MA 02215, USA; 2 Ministry of Fiscal Property, Av. Bdo O’Higgins 720, Santiago, Chile; E-Mail: j.gabriel.fernandez@gmail.com

**Keywords:** risk adjustment, risk selection, health care system

## Abstract

Interest has grown worldwide in risk adjustment and risk sharing due to their potential to contain costs, improve fairness, and reduce selection problems in health care markets. Significant steps have been made in the empirical development of risk adjustment models, and in the theoretical foundations of risk adjustment and risk sharing. This literature has often modeled the effects of risk adjustment without highlighting the institutional setting, regulations, and diverse selection problems that risk adjustment is intended to fix. Perhaps because of this, the existing literature and their recommendations for optimal risk adjustment or optimal payment systems are sometimes confusing. In this paper, we present a unified way of thinking about the organizational structure of health care systems, which enables us to focus on two key dimensions of markets that have received less attention: what choices are available that may lead to selection problems, and what financial or regulatory tools other than risk adjustment are used to influence these choices. We specifically examine the health care systems, choices, and problems in four countries: the US, Canada, Chile, and Colombia, and examine the relationship between selection-related efficiency and fairness problems and the choices that are allowed in each country, and discuss recent regulatory reforms that affect choices and selection problems. In this sample, countries and insurance programs with more choices have more selection problems.

## 1. Introduction

Social welfare concerns are part of every modern health care system, where issues such as coverage for the poor and unequal access are often discussed. By nature, the distribution of health care costs is highly skewed as a large share is spent on relatively few people. Not only is actual spending skewed, but expected spending is also highly skewed: some consumers are predictably more expensive than others. The predictability of heterogeneous risks can lead to risk selection as agents try to take advantage of private information to attract profitable enrollees. Risk selection is exacerbated in the presence of income inequality, which affects consumer’s ability to pay, and helps explain the need for some level of solidarity in every health care system design. Expanding on the definition of Newhouse [1], we define **risk selection** as: “the outcomes of any choice process in which any of the four agents (consumers, providers, health plans, sponsors) exploit unpriced risk heterogeneity and break pooling arrangements”. Below, we describe the efficiency and equity problems that arise from risk selection, and give examples of the selection tools that each agent might use to influence choices.

In this work we present a conceptual model that helps understand risk selection and how risk adjustment and risk sharing are used to address it. **Risk adjustment** refers to the use of information to calculate the expected health care expenditures of individual consumers over a fixed interval of time (e.g., a month, quarter, year) to set payments between agents; **risk sharing** implies some type of retrospective payment of agents for some fraction of their ex post costs. Risk sharing and risk adjustment often work together and can take place between any of the four agents to reallocate resources. Two solidarity dimensions often motivate risk adjustment and risk sharing: income solidarity and risk solidarity [2], where solidarity implies a separation of ability to pay from need for health care services but resulting in ex ante cross subsidies. **Income solidarity** implies that high-income consumers should subsidize low-income consumers, while **risk solidarity** implies that low-risk (healthy) consumers should subsidize high-risk (sick) consumers. The importance attached to each type of solidarity varies across countries.

In order to avoid confusion, in the next section we develop an overall conceptual framework and define the terminology relating to the four main decision-makers in health care markets: the consumer, the sponsor, the health plan and the provider.

A second section of this paper contrasts the experiences of four countries in the Americas-Canada, USA, Colombia and Chile. We choose these four countries for convenience, but also because they provide appropriate comparisons and contrast for our framework. Each country is reviewed while attempting to do three things. First, we use the conceptual framework from the previous section to characterize different insurance systems in a systematic way, identifying the four broad classes of agents in each system. Second, we highlight the choices each agent must make and the instruments or tools available that affect these choices while reviewing the financial and contracting relationships between the different agents. Differences in the choices that are allowed turn out to help our understanding of why the selection experience varies so much across different countries. Third, we briefly summarize how countries are using regulation, risk adjustment and risk sharing to improve selection-related problems. After describing the experience of four countries in our study, we end with concluding thoughts and a brief policy discussion of recent changes and proposals.

## 2. Conceptual Framework and Definitions

**Figure 1:**
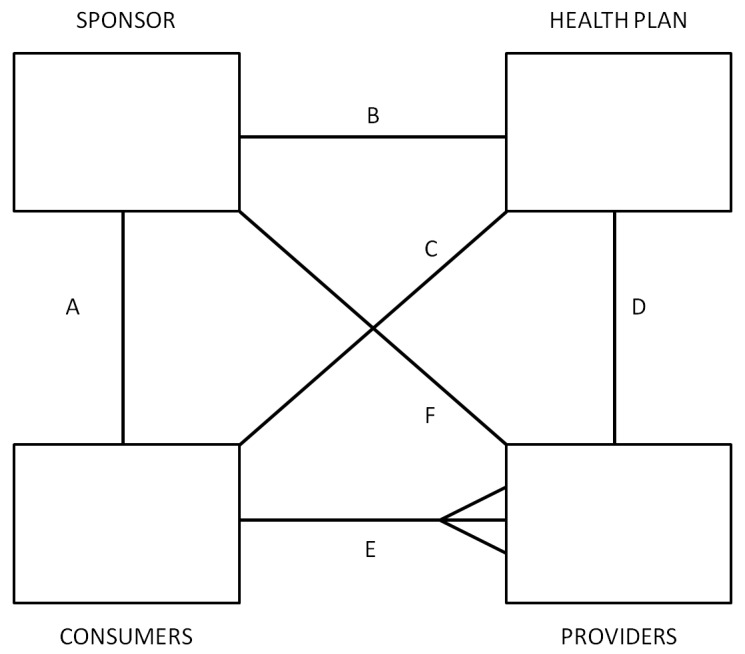
General framework.

In order to understand the rationale for risk adjustment and risk sharing, it is important to understand the selection and incentive problems that are to be corrected. Figure 1 illustrates the four fundamental decision making agents in health care markets and the possible contractual relationships between them: consumers, providers, health plans and sponsors.

**Consumers** receive health care services and choose which health plan to purchase. In some settings (e.g., Canada, Netherlands, US Medicare) consumers are individuals or members of the same family who opt for different health plans. In other settings (e.g., US private insurance, Germany, Japan), the household is constrained to choose the same health plan. In some instances, insurance features such as family deductibles integrate consumption choices of multiple individuals.

The second broad agent type, **providers**, is the party that supplies health care services. This category includes doctors, hospitals, and pharmacies selling prescription drugs.

The third type of agent, **health plans**, is fundamentally responsible for paying and contracting with providers. In some settings, health plans may be able to selectively contract with providers, or consumers and sponsors. In the US, health plans offered by health insurance companies may include traditional indemnity plans, preferred provider organizations (PPOs), and health maintenance organizations (HMOs) while in Europe they are often called the “sickness funds”. Note that health plans need not actually provide insurance, for instance they may provide administrative services only, and pass the risk on to another agent.

Finally, the **sponsors** are intermediaries who are willing and able to redistribute ex ante expected financial cost of health care across consumers and among health plans. The crucial role of the sponsors is that the payment received by a health plan for a given consumer need not be the same as the payment made by that same consumer. For example, the sponsor may charge consumers in proportion to their income, but pay health plans according to the expected cost of their enrollees. Or the sponsor may contribute a fixed amount to each health plan, and regulate the premiums that health plans charge to individuals and families. To identify the sponsor in a health care system, we ask the question: who keeps the insurance premium contribution paid for a 60 year old unhealthy worker from being at least ten times higher than that of a 20 year old healthy worker? In many countries employers or coalitions of employers serve the role of being sponsors, while in others governments or independent agencies perform this role. Despite the incentives to risk select, health plans can also be sponsors in some instances (with mandatory community rating).

Two other important classes of agents in most health care systems are insurers and regulators. An **insurer** is an entity who in the current contract period bears the financial risk of spending an extra dollar on health care; and a **regulator** is an agent that sets the terms of competition, defining possible choice options, and restricting possible actions by all of the other agents. The insurer is not defined as an independent agent in our framework because any of the four agents can potentially share the role of being the insurer. Some consumers self-insure in the US (those without any insurance), and demand-side cost sharing imposes financial risk on consumers. Sponsors such as government agencies and large employers often self-insure (e.g., Canada, U.S. federal employees, and U.S. Medicare). Health plans (such as HMOs in the US, or private insurance plans or sickness funds in Europe), or even providers may also provide insurance (such as GPs in the UK, or some group practices that accept capitated payment in the US). The role of the insurer is often shared among several agents, and can be found by asking who in the current contract period bears the marginal cost of one more office visit, inpatient admission, or outpatient drug.

As with insurers, a regulator can also be a sponsor (government or employer), health plan organization, provider organization, or even a consumer organization. Part of this role is always played by the government (by setting the rules of the playing field for the health care market to develop). Other common institutions that share the role are associations of providers (e.g., Germany, Canada, and the Netherlands). These associations may standardize fees and regulate provider behavior. In some cases, provider associations also redistribute provider payments by limiting aggregate payments to individual physicians (Germany), or bearing some financial risks (Canada).

In some countries the roles of two or more of these agents are combined into one agent, which implies that this agent internalizes the relevant decisions. We discuss this arrangement below when discussing Canada.

The six solid lines connecting the various agents in Figure 1 signify contractual relationships, which may imply both financial payments, as well as contractual terms. The classic relationship is for sponsors to collect revenue from consumers, and then make payments to health plans, who make payments or otherwise contract with providers. There is also a special contract between consumers, who receive services from health care providers, and health plans, which pay for such services. The contractual relationship that is used the most rarely is the direct link between sponsors and providers; we have left it in to capture the possibility that the sponsor may contract with some or all providers separately from health plans, such as by paying performance bonuses that cut across multiple health plans.

An innovation to the usual depiction of these four agents is that in addition to showing the contracting relationship between the various agents, we also indicate whether an agent has choice in which agents to contract with. Selection problems arise in instances where an agent has choices. Reflecting the idea of a decision tree, we use multiple line segments fanning out between different agents to signify choice and hence the possibility of selection problems. Hence in Figure 1, consumers are shown as having a choice of providers, and potentially this can cause selection problems. If providers are all paid fee-for-service (FFS), then they will be relatively indifferent as to whether they treat a more or less sick set of consumers. However if they are paid on a lump sum basis, such as by salary, capitation, or some bundled payment such as diagnosis related groups (DRGs), then providers will not be indifferent, and will have a selection incentive. Whether selection is a problem depends upon the choices agents have available, the information they possess, what tools are available for selection, as well as regulatory, legal and ethical constraints.

Depending on the country, each of the contractual relationships shown in Figure 1 may involve elements of choice: all except the one between sponsors and providers are relevant in the US, for instance. We attempt here to briefly highlight some of the recent literature developing theoretical models about each of these linkages.

### Linkages between the Four Agents

**Linkage A** is between the consumer (individuals or families) and their sponsor (who may be a government agency, employer or other independent organization). Only when the sponsor is the employer is there likely to be a choice of sponsor by consumers. Employment-based health insurance introduces choice along linkage A, and has been researched extensively for its impact on health care markets. For an excellent review, see Gruber [3]. In the U.S., one line of research has examined “job lock” and other inefficient labor market choices [4], while another line of research concerns employer willingness to offer insurance and the use of premium cost sharing to influence employee health plan choices [5]. Theoretical models include Crocker and Moran [6] and Dey and Flinn [7]: the first analyzes the role of health insurance as a commitment device, while the second uses a matching model to analyze the economic welfare implications of job mobility and its interaction with health insurance. Discrimination by age and discrimination against persons with chronic illnesses or disabilities are further examples of “selection problems” as we define it here. Ellis and Ma [8] study the effect of labor market turnover on employers’ willingness to offer health insurance.

**Linkage B** is between the sponsor and the health plan or plans. The decision to offer insurance, risk adjustment and risk sharing, and choice of plan types offered by sponsors are all relevant to this choice. The huge risk adjustment literature has primarily focused on changing incentives with this linkage. Some developments in this area are touched on briefly below.

**Linkage C** is a direct link between the consumers and the health plans. This linkage is relevant when consumers are permitted to choose health plans from options offered by their employer or other sponsor. Risk adjustment and risk sharing are rarely used to change linkage C. Indeed in most countries the sponsor imposes rate restrictions on the consumer’s direct premium contribution to the health plan. In this way risk solidarity if not income solidarity is ensured so that high expected health care cost consumers pay the same premium as low expected health care cost consumers.

Linkage C is also a mechanism relevant for choices by plans to avoid certain individual enrollees. Note that the plans’ incentives for risk selection largely stem from the unpriced risk heterogeneity created by the rate restrictions imposed by the sponsor or by self regulation (perhaps intended to prevent sponsor regulation). The most extreme form of risk selection along this dimension is dumping by health plans, whereby plans explicitly deny coverage to unprofitable consumers. Health plan exclusions for preexisting conditions are another mechanism used here. Shen and Ellis [9,10] develop an explicit model of risk selection of this type, and explore how well existing risk adjustment models reduce the profitability of risk selection in a sample of Medicare individuals. They do not argue that plans are able to actually achieve this level of risk selection, only that it is interesting to understand the potential magnitude of the problem. They find that risk adjustment only modestly reduces the profitability of selection, and that better risk adjustment models and weaker information by health plans reduces the selection problem. Buchmueller [11] evaluates the effect of reforms aimed at reducing age selection in Australia, showing that in private markets financial incentives can increase enrollment of otherwise uninsured young people. A more recent literature has focused on the information asymmetries and how reporting can have an impact on each agent’s decisions [12].

**Linkage D** is the direct link between health plans and providers. It is often the most complex. Provider payment and case management, selective contracting, as well as plan initiated service distortion to attract profitable enrollees are all relevant to this linkage [13]. Risk adjustment is sometimes used within plans to transfer risk (insurance) onto providers groups or even individual providers. There is a rich literature that explores this issue, Newhouse [1] being a classic. McGuire [14] provides an especially useful overview of physician reimbursement incentives and the agency problems to be corrected, while Pauly [15] discusses balance billing and focuses on classic trade-off of correcting moral hazard and risk aversion problems. A more recent literature has focused on separating the role of the primary care providers and more complex care providers to control cost pressures [16], where payment mechanisms can help aligning incentives and promote prevention. A large discussion is taking place in the business press and policy circles on this issue [17].

**Linkage E** is the direct linkage between consumers and providers. Demand-side cost-sharing, waiting times, provider convenience, amenities, and provider quality all may affect choices made at this level. Health plans may restrict consumer choice of providers through utilization controls or through selective contracting. All of these benefit plan features will influence selection decisions if they vary across health plans. Examples include the “Medical Savings Accounts” (MSAs) [18] and “consumer driven health care” [19], which often entail plans with high deductibles. These approaches revitalized interest in demand-side incentives, including some inequities and selection problems associated. Another approach has been to focus on the lack of information about provider’s quality. Dranove *et al.* [20] show how the existence of report cards can address information asymmetry related problems.

**Linkage F** is the direct linkage between a sponsor and providers. Cases where this is used are rare, but include the US Medicare program’s “assignment program”, whereby physicians receive a lower fee if they do not contract to accept the Medicare fee schedule as payment in full for their services. Other examples include incentive systems in which a government or employer agrees to pay a bonus or other performance payment to a provider, which might include patients covered by multiple health plans. We omit this linkage on diagrams where it is not currently used.

## 3. Risk Selection Problems

Health economists come up with a very wide range of problems that are attributed to risk selection, and the problems identified can vary enormously across countries. In Table 1, we attempt an exhaustive list of selection problems and divide them up into efficiency and equity problems. We focus here on two dimensions to categorize the problems: the inefficiencies and inequities of the outcomes of risk selection, and also on the inefficiencies that are a direct consequence of those actions aimed at breaking pooling arrangements, irrespective as to whether these actions (on balance) are successful. While most outcome-related problems are relevant for linkage C between consumers and health plans, similar problems may occur in linkage E between providers and consumers if the provider is paid via capitation, a lump sum budget, or DRG-payment (except the “group access problem”, which is associated with linkage B between sponsors and health plans). Action-related problems are more likely relevant for linkages A (labor market problem), D and E (service distortion problem).

**Table 1:** Summary of perceived selection problems in different health care systems.

Problems	Alberta Canada 2012	US Medicare 1985	Chile Public 2012	Colombia 2012	US Medicare 2012 *^a^*	Chile Private 2012	US Private Employers 2012 *^a^*
**What selection problems are considered serious?**							
*Efficiency Problems*							
Incomplete insurance—Consumers bear too much risk		X		X	(X)	X	(X)
Individual access—Can individuals always find a “fair” plan?							(X)
Group access—Can employers always find a “fair” plan?					X	X	(X)
Service distortion problem—Too much or too little of some services			X	X	X	X	X
Wasted resources—Too much advertising or administration				X		X	(X)
Labor market problems—Job frictions							X
Patient sorting problem—Providers sort patients, offer different quality			X	X	X	X	X
Waiting time problem—Plans use waiting time to ration care	X						X
Plan turnover problem—Consumers forced to change plans too often					X	X	X
*Equity Problems*							
Risk solidarity problem—High risks pay too much for health insurance						X	(X)
Income solidarity problem—No subsidy from high to low income consumers		X			X	X	(X)
Free rider problem—Some people choose not to be insured				X *^b^*			(X)
Plan over/underpaying problem—Plans paid too much/too little			X		X	X	X
Provider over/underpaying problem—Providers paid too much/too little	X	X	X	X	X	X	(X)
Simple Count of X’s	2	3	4	6	8	10	14

Notes: Items reflect subjective valuation by the authors. *^a^* Items in parenthesis were addressed by the 2010 reform, although not necessarily eliminated; *^b^* Choosing not to be insured is illegal, but there is an enforcement problem.

The first efficiency problem is the classic Rothschild and Stiglitz [21] problem: consumers with heterogeneous risks may have difficulty finding a health plan willing to offer them the amount of insurance that they desire, and hence may end up being imperfectly insured. Dependent upon the level of the contracting costs either the low-risk individuals or the high-risk individuals cannot obtain as much health plan coverage as they wish at their actuarially fair price [1]. We label this the “**incomplete insurance problem**”. This outcome-related problem has both efficiency and equity implications, but we have chosen to list it as an efficiency problem. This can occur because plans deny access, discontinue insurance contracts, or exclude people with certain pre-existing conditions from coverage. Or it can result when consumers react to plan features and sort themselves into different plans. Glazer and McGuire [22] make a further distinction between two closely related forms of this access problem: the **individual access problem** and the **group access problem**. In the latter case agents such as employers, health plans or providers avoid whole groups of consumers, such as a geographic area, an occupation or an employer because they are viewed as unprofitable.

The fourth efficiency problem (the first action-related one), is the **service distortion problem**, whereby health plans or providers distort the quantity or quality of service so as to attract a relatively profitable set of consumers [1,13,23]. This occurs if health plans have a disincentive to be responsive to the preferences of the high risks because the high risks are unprofitable consumers e.g., due to premium rate restrictions and inadequate risk adjustment.

Another efficiency action-related problem is the **labor market frictions problem**, which is the problem of inefficient labor market choices such as the “job lock” [3,4]. In many countries with employment based insurance, consumers may be unwilling to retire, change jobs, or leave public insurance systems because of fear of losing health insurance.

Since selection activities are often more profitable than managed care activities, in the short run —when a health plan has limited resources available to invest in cost—reducing activities-it may prefer to invest in risk selection rather than in improving the efficiency in the production of care, which we call the **incentive problem**. Ellis and McGuire [23], Glazer and McGuire [24], Frank *et al.* [25] develop rich models of this selection strategy. Finally, if selection actions produce no social gain, all resources devoted to it can be considered a **waste of resources**. This holds for the resources devoted to service distortion (including patient sorting), or the transaction costs of the low-risk individuals who will persistently (try to) separate themselves from the high-risk individuals by buying new products that are especially designed to lure them from the more heterogeneous risk pool (even through long waiting lists). Also the continuous exit and re-entry of health plans have real social costs.

The first equity problem listed is at the heart of the US debate over health care reform: How much concern is appropriate about high risks bearing much high costs than low health risks? This risk-solidarity problem figures prominently in Europe, Canada and many other countries. This concern, rooted mostly in welfare systems, is usually confounded with a second equity problem: income-solidarity. Both are related to income selection and risk selection. The underlying idea is that low income people will present higher risks [26,27]. Ter Meulen and Jotterand [28] point out the trend of European health delivery system moving towards two-tier health care system, with emphasis on individual responsibility along with solidarity. The objective of this reconstruction is to control health cost as well as maintain equal access to health care and suppress selection problem. Despite the discussion, successful selection actions result in a market segmentation whereby the high risks pay a high premium and the low risks pay a low premium. Many policy makers and consumers in Canada, Europe and elsewhere consider this lack of risk solidarity a social problem. This solidarity problem that results from selection is closely related to the solidarity problem that results from explicit premium differentiation. The selection problem occurs if health plans are not able (because of high transaction costs) or not allowed (because of premium rate restrictions set by a regulator or by self regulation) to let the premium fully reflect expected costs.

A further equity problem is the **free-rider problem**. Free riders are (usually low-income) people who intentionally do not buy health insurance because they anticipate that others in society will pay if they really need expensive care.

Yet another common equity problem due to market segmentation is an **overpaying/underpaying problem** of health plans [29,30]. For example, the adversely selected health plans may go bankrupt because of underpayment. Consumers of these plans may miss the reimbursement of their health expenses, and may have a hard time finding a new health plan. Another consequence may be that efficient health plans that do not risk select consumers are driven out of the market by inefficient plans that are successful in preferred risk selection. And if the sponsor pays the adversely selected market segment on a fee-for-service basis (as Medicare does in the US), the sponsor may incur a financial loss due to the selection.

A complication with solving the selection problems is that some tools to reduce outcome-related selection problems increase the action-related selection problems. For example, forbidding the exclusion of pre-existing conditions from coverage or requiring guaranteed continuity-of-coverage or a periodic open enrollment increases the service distortion problem, because forbidding direct selection increases the health plans’ incentive to use the more subtle tools for risk selection, such as service distortion.

### Choices That Can Lead to Risk Selection

Table 2 provides a summary of the potential choices that each of the four agents might have at his disposal. Each time a choice is allowed, it has the potential to encourage risk selection problems.

The **consumer** has potential tools for selection if he has a choice of sponsor, a choice of whether to be insured (and for what coverage), choice of health plan, and choice of provider. Sometimes these choices are associated with geographic choices (e.g., in Canada, health insurance depends on the regional authority).

The **sponsor** has potential tools for selection if he has a choice of whether to offer coverage and how much coverage to offer. Also the choice to prioritize some services (giving different levels or urgency) can become a selection tool. The freedom to choose enrollees (e.g., an employer choosing its employees) in employer-based health care systems is also a tool for risk selection. Other relevant tools sponsors have are whether to offer personal or group/family coverage, and how the additional costs of dependents are to be shared between the enrollee and sponsor. A general tool is the payment mechanism chosen to contract with health plans and how much of risk sharing and risk adjustment will be used to contract with plans.

**Health plans** have a great variety of potential tools for selection, which include the selection of services to cover; demand side cost sharing (e.g., deductibles) and their design; selective contracting with certain but not all providers of a given type; forms of provider payment, which give providers incentives for selection (e.g., risk sharing between the health plan and the providers); selection of geographic markets; excluding preexisting conditions; denying coverage; underwriting; selective advertising; and by offering a package deal of health insurance and other forms of insurance services bought mostly by relatively healthy people, including supplemental health insurance or fitness club membership (“tie-in sales”).

**Providers** of care also have many tools for risk selection. The most obvious one is to refuse to treat certain patients, or to refer more complex, expensive, or unwanted cases to other providers. Other more subtle tools for risk selecting patients include differentiated waiting times for the different types of services so as to attract profitable patients; selecting fee schedules for treating uninsured patients; and the use of any patient sorting mechanisms for access to treatment. Providers can also risk select via the advice of the physician-gatekeeper (although gatekeepers can also have positive efficiency effects associated mostly through the rationing element of care). Providers can sometimes choose which health plans they will affiliate with, which extends the plan risk selection to themselves. Providers may also be able to choose how much “balance billing” above the covered charge they will require for different types of patients and health plans.

**Table 2:** Summary of choices available in different health care systems.

Choices	Alberta Canada 2012	US Medicare 1985	Chile Public 2012	Colombia 2012	US Medicare 2012 *^a^*	Chile Private 2012	US Private Employers 2012 *^a^*
**Which choices are available to each agent?**							
*Sponsor*							
Choice not to offer insurance?							(X)
Choice of health plans?					(X)	X	X
Choice of benefit features?			X	X	X	X	X
Choice of premium cost sharing?			X	X	X	X	X
Financial reward for reduced coverage?					X		X
Choice of premiums varying by income?			X	X		X	X
Choice of premiums for family *vs*. individual coverage?						X	X
Choice of pay-for-performance incentives?			X	X			X
Choice of risk adjustment?	X	X		X			X
*Health Plan*							
Choice of benefits to offer?			X		X	X	(X)
Choice of demand side cost sharing to consumers?			X	X	X	X	X
Choice of providers with whom to selectively contract?			X	X	X	X	X
Choice of provider payment?			X	X	X	X	X
Choice of geographic area to serve?				X	X	X	X
Choice of performance measures to providers?			X	X	X	X	X
Is exclusion of preexisting conditions allowed?					X	X	(X)
Is underwriting allowed (denying coverage)?					X	X	(X)
Is direct advertising allowed?				X	X	X	X
Tie-in sales of alternative insurance policies allowed?					X		X
*Provider*							
Choice of patients when at less than full capacity?			X	X	X	X	X
Choice of balance billing?			X *^c^*	X	X	X	X
Is there a primary care gatekeeper?	X		X	X	X		X
Choice of specialists without a referral?				X	X	X	X
Choose of different patient waiting times?	X		X	X	X	X	X
Can a hospital refuse to treat if no coverage?						X	X
Patient sorting across hospitals and doctors?			X	X	X	X	X
*Consumers*							
Choice of sponsor?							X
Choice of whether to be insured?				X *^b^*	X		(X)
Choice of health plan?				X	X	X	X
Choice of which family members to insure?						X	(X)
Choice of different benefit feature?					X	X	X
Choice of primary care provider?	X	X	X	X	X	X	X
Choice of specialist?	X	X	X	X	X	X	X
Simple Count of X's	5	3	16	21	26	24	32

Notes: Items reflect subjective valuation by the authors. *^a^* Items in parenthesis were addressed by the 2010 reform, although not necessarily eliminated; *^b^* Choosing not to be insured is illegal, but there is an enforcement problem; *^c^* Limited by fee schedule.

## 4. Correction of Risk Selection Problems

### 4.1. Risk Adjustment and Risk Sharing

Risk adjustment and risk sharing are commonly used to address risk selection issues. Risk adjustment refers to the use of information to calculate the expected health care expenditures of individual consumers over a fixed interval of time (e.g., a month, quarter, year) and to set payments between agents; risk sharing implies some type of retrospective payment of agents, usually of some fraction of their ex post costs. Both risk sharing and risk adjustment usually work together and can take place between any of the four agents (see Ellis and Layton [31] for a recent review of the developments of risk adjustment).

The most ambitious literature on risk adjustment has modeled multiple linkages between agents simultaneously. An important set of papers, by Glazer and McGuire [24,32], develop the concept of optimal risk adjustment, which attempts to offset selection incentives. Central to their model is the idea that managed care plans, which are an integrated agent that encompasses health plan and provider decisions, are able to ration services selectively so as to influence the attractiveness of their plan to consumers. They explore how the sponsor should change the risk adjustment payment formula given that health plans and consumers may have private information to use for making health plan enrollment choices. They characterize how conventional risk adjustment will be sub-optimal given selection behavior and define how signals should optimally be made. Frank *et al.* [25] operationalize the concept of optimal risk adjustment by providing empirical method on US Medicaid data for quantifying the magnitude of the selection problem using variance and covariance terms. Ellis and McGuire [23] explore the distinction between the predictability of various services and their predictiveness. They illustrate using US Medicare data that service distortion using annual spending by type of service is more effective for health plans than service distortion of spending by diagnosis or provider type, largely because spending by type of service is more highly predictable, *i.e*., able to be anticipated, than spending on specific providers or diseases. Jiang *et al.* [33] verify that risk adjustment removes about half of the incentive to select by distorting service offerings at the plan level.

A new form of risk sharing arises when a concurrent (also called retrospective) risk adjustment model is used in which diagnoses or health conditions from a given year is used to calculate payments for that year. Unlike a prospective model that uses ex ante health status information, concurrent models can recognize new health conditions (broken arm, heart attack, a new pregnancy, or case of the flu) and compensate plans ex post for the acute costs of these conditions.

Although risk sharing is a very common topic in development economics and in models of consumption and labor at the family level, there is a more modest literature on the role of risk sharing in reducing health plan level selection. Marchand *et al.* [34] develop a theoretical model of plan level risk sharing and argue for the use of lagged health spending as a simple risk adjuster/risk sharing tool to reduce selection. Garcia Goni [35] incorporates both risk-sharing and risk adjustment in a rigorous theoretical model and derives conditions under which the social optimum is and is not achievable. Risk sharing has received more attention in Europe than in the US, although US employers often do choose between self-insuring, partially or fully insuring the health risks facing their employees [36].

### 4.2. Regulation and Risk Selection

Risk adjustment and risk sharing are only two of the policy tools available to influence risk selection. In all countries a wide array of regulations on the choices and structure of the system also affect selection. Table 3 summarizes many of these “techniques” or choices that can be regulated that influence selection. Of course, each of these measures may also have unexpected consequences on efficiency or quality of care. In this section we highlight some forms of regulation and how they affect selection.

**Table 3:** Summary of techniques available that influence selection in different health care systems.

Techniques	Alberta Canada 2012	US Medicare 1985	Chile Public 2012	Colombia 2012	US Medicare 2012 *^a^*	Chile Private 2012	US Private Employers 2012 *^a^*
**Which techniques are available to increase/reduce selection?**							
*Consumers*							
Choose not to become insured until high health costs				X *^b^*			(X)
Choose low benefit plans until needs become great					X	X	(X)
*Providers*							
Undertreatment of high cost patients				X	X	X	(X)
Underprovision of services used by high cost patients			X	X	X	X	X
Recommendations to patients to change plans or providers					X	X	X
Delaying visits by high need patients			X	X	X	X	X
*Health plans*							
Selective advertising				X	X	X	X
High deductibles and copayments that deter high cost patients					X	X	(X)
Differential enrollment based on consumer survey results					X	X	X
Exclusions for preexisting conditions						X	(X)
Genetic testing and use of information to enroll						X	X
Charging higher premiums for high health cost enrollees						X	(X)
Shortage of specialists contracted with			X	X	X		X
Delayed payments affect high cost enrollees			X	X	X	X	X
*Sponsor*							
Risk adjustment (bundled payment, set up ex ante)				X	X	X	
Risk sharing (ex post)				X	X		
Report cards and consumer information				X	X		X
Benefit plan features variation					X	X	X
Premium cost sharing (how premiums vary across consumers)						X	(X)
Premium variation by income				X		X	(X)
Definition of family for family coverage			X			X	(X)
Premium rate bands (levels or rates of increase)			X		X	X	(X)
Supplementary insurance features.	X	X	X	X	X	X	X
Ease of referrals					X		X
Selective contracting in geographic areas with low cost populations				X	(X)	X *^c^*	X
Simple Count of X's	1	1	7	12	18	19	23

Notes: Items reflect subjective valuation by the authors. *^a^* Items in parenthesis were addressed by the 2010 reform, although not necessarily eliminated; *^b^* Choosing not to be insured is illegal, but there is an enforcement problem; *^c^* Urban *vs*. rural, based more on private providers availability than low risk.

Regulation can exist on any of the dimension of choice presented above. The first type of regulation is what kind of institution is allowed in the health care market: public or private, not for profit or for profit. The ownership of these institutions will have an impact on risk selection as a non-profit institution may be more willing to accept bad risks, or may limit the ability of private or for-profit health plans to extract surplus from the “good” risks. A closely related issue is whether vertical integration is allowed or not. Whenever vertical integration is allowed, forced or encouraged, some dimensions of choice are absorbed by the same agent limiting selection. The extent of competition, as opposed to cooperation, also has an impact on available resources, and therefore on the specialization of different agents which impacts selection incentives. Finally, regulation can also determine which agents will serve the insurer’s role and how risk is shared between agents, by either regulating payments or interactions between agents of the same type.

Regarding provider regulations, some systems allow only public providers (e.g., hospitals in Canada), so that feasible choices are likely to depend on the region in which the consumer lives. This restriction limits many dimensions of choice for consumers and therefore, selection problems although some would argue that this creates an inadequate incentive for high quality. More common in systems with public delivery is to permit both public and private providers (e.g., Australia, Chile, Colombia), giving consumers the option between physicians with different incentives. Regulation of what private providers are allowed and not allowed to do, and in particular of how public physicians can also treat private patients [37], can greatly affect provider’s ability to risk select. Provider regulation can also restrict fee schedules and/or payment mechanisms, including establishing bundled payment or risk adjusted payments for certain conditions. Differentiating primary care from more advanced and complex care, limiting referrals or imposing protocols for them is another measure potential regulators can implement to regulate provider’s actions. A very common regulation on providers limits their ability to turn away emergency cases, which generates very complex incentives on ER usage by patients.

Health plan regulation often limits the tools of selection named in the previous section such as whether they are allowed to have preferred providers, transparency on the negotiated fees, cream skimming, dumping, premium regulations, among others. For sponsors the law will usually determine a minimum coverage they must provide, as well as the treatment of unemployed people and their dependents. For consumers, regulations include laws requiring individuals to purchase health insurance and how often consumers can switch between plans.

## 5. Lessons from the Americas

This section contrasts the experiences of four countries in the Americas -Canada, Colombia, Chile and the US -in dealing with selection. This section not only presents the way different countries use risk adjustment and risk sharing in different settings, but also provides an example of how the previous analytical model can be used for presenting different organizational structures emphasizing the role and interactions of the four broad classes of agents identified in the model (see Ellis *et al.* [38] for a complementary approach comparing among developed countries).

One of the challenges facing an overview paper such as this is that the country systems being described are constantly changing. This overview attempts to capture the institutional arrangements in place as of December 2012, using both the generic terminology from the first section, and country-specific names and institutions.

Rather than presenting the countries in alphabetical order, we have chosen an order that we find more informative and leading to useful contrasts. Also, we have divided up US and Chile into multiple descriptions of the fundamental agency relationships to capture their complexity. At the end of this section we present a synthesis and summarize lessons for other countries with similar problems and proposals for reform.

### 5.1. Canada

Although the Canadian Health Act mandates that every Canadian be insured, the regulation and financing of health care in Canada is the responsibility of the 13 provinces and territories. None of the provinces or territories in Canada offers multiple competing health plans, which largely eliminates plan level selection incentives. Despite this, a slight selection problem still exists, arising from consumer choice of residence, and consumer choice of providers. Canada is also interesting in that its health care system is similar to certain other countries with a social insurance program, including countries as diverse as Australia, North Korea, Norway, and Taiwan. Health system financing, the list of covered services and delivery systems in Canada vary across the provinces. We focus here on Alberta, which has excellent data and is among the furthest along in worrying about fairness and selection issues.

#### 5.1.1. Alberta’s Health Care System

**Figure 2:**
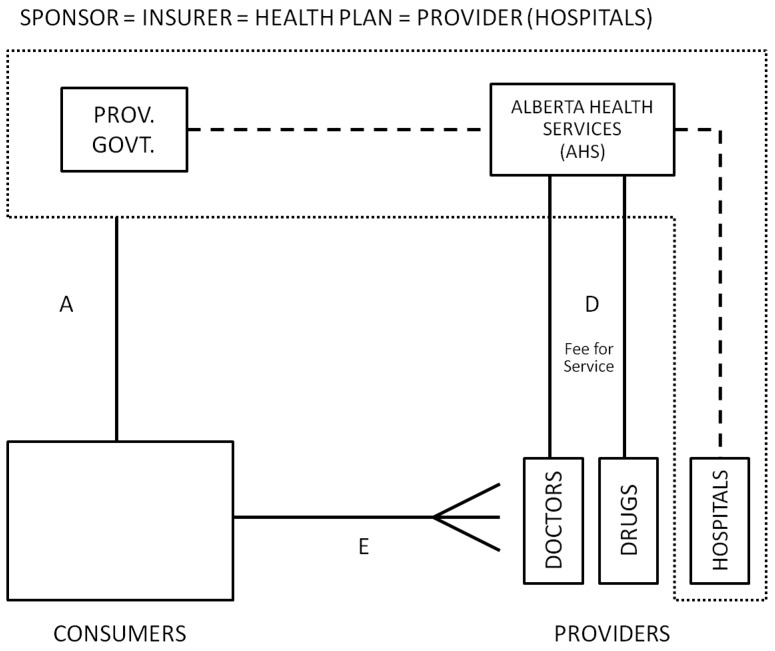
Alberta, Canada.

A stylized view of Alberta Canada’s health care system is depicted in Figure 2. All residents are automatically covered by the province in which they reside, and payments are collected from workers and employers through mandatory social insurance premiums and general income taxes. The provincial government ministry of health, called Alberta Health and Wellness (AHW), pays for most physician and office-based services directly through a provincial wide fee schedule. Hospital and facility based health care is organized under the Alberta Health Service (AHS), which is responsible for spending on hospitals, health facilities and home care. Until 2008, there were 17 regional health authorities (RHAs) and a provincial cancer board with significant autonomy in allocating their budget to hospitals and facility-based care. In 2008, these RHAs were merged into a unique provincial entity called AHS. Alberta residents are provided with full coverage for medically necessary health procedures through the Alberta Health Care Insurance Plan (AHCIP). Drugs not administered in hospitals are not included as a benefit for all residents, but are covered for those over age 65 under a co-pay arrangement. The government also sponsors an additional prescription drug plan for those under 65, but residents are allowed to opt out. The nonprofit firm Alberta Blue Cross is contracted to process drug claims, which are paid on a fee-for-service basis. Consumers are insured as families or individuals and pay low co-payment fees for some services. Consumers are free to choose to visit any primary care practitioner, although a referral is generally needed to see a specialist. In addition to this publicly funded system, consumers as individuals or with sponsorship from their employers are able to purchase supplementary insurance that covers pharmacy costs and limited other uncovered services, but these policies are not allowed to replace or augment coverage of services already covered by the public system (Supplementary insurance is inherently subject to selection bias given that it is voluntary.).

#### 5.1.2. Selection Problems

Given that there is only one health plan, and that providers are paid primarily on a fee-for-service basis, it would seem that there are no opportunities for selection problems to emerge. This is not exactly true. Although selection problems are small in comparison to other countries, they still occur in two ways. First, residents do not choose where to live randomly. Second, the AHS still has to deal fairly with patient sorting across health care facilities, ensuring fair payments to each facility given the case mix of patients that they manage, treat or refer.

AHW has explored this geographic selection bias, and finds evidence of it [39]. In particular, they find persons with more serious chronic illnesses are more likely to live in urban areas, and that certain diseases vary significantly across regions. Geographic variation within Alberta, in terms of urban *versus* rural, and distances from rural areas to urban hospitals are probably greater than in many other countries (such as the Netherlands) that are more homogeneous in their access. Nonetheless, the Alberta experience reminds us that consumers do sort themselves geographically in meaningful ways (Geographic sorting has been mentioned as a concern by others, and has important implications for work such as that of McClellan and Newhouse [40] and others that uses distance to hospitals as an exogenous instrument for access.).

#### 5.1.3. Role of Risk Adjustment and Risk Sharing and Plans for the Future

The Alberta provincial government is the insurer and health plan for physician services. The total budget for physician expenses is negotiated annually with the Alberta Medical Association (AMA), a professional association representing 95% of Alberta physicians. The AMA negotiates three things with AHW: (1) the schedule of medical benefits to be covered and the level of cost sharing and fees for them, which are negotiated only occasionally but fine tuned frequently; (2) the annual increase in schedule prices, negotiated with two-to three-year contacts; and (3) the total adjustable cap on all fee-for-service expenditures (province-wide and not specific to individual patients or practitioners). This cap is negotiated annually, and AHW and the AMA in principle share the risk once the cap is reached. In theory, the AMA physicians assume all risk (work for free) if this cap is hit and not adjusted. Similarly, the provincial government is the insurer for pharmacy expenses of persons over age 65 and bears the full expenditure risk. The AHS bears the risk for inpatient and other services that fall in its jurisdiction.

Until 2002, Regional Health Authorities (RHA) budgets were primarily allocated using demographic risk adjuster. Later on, more complex risk adjustment methods were studied. However, after the 2008 reforms that unified budget decision making the need for risk adjustment across regions was eliminated. Within this framework, the biggest role for risk adjustment is for paying providers. Moreover, reports like *Economic Policy Reforms 2010* [41] are promoting different payment methods for primary care, including mixed remuneration system for primary-care providers [42]. If services are provided by associations that are big enough to bear some risk, then payment methods should be risk adjusted to include the risk of their patient mix.

### 5.2. United States: Medicare

#### 5.2.1. Health Care System Overview

**Figure 3:**
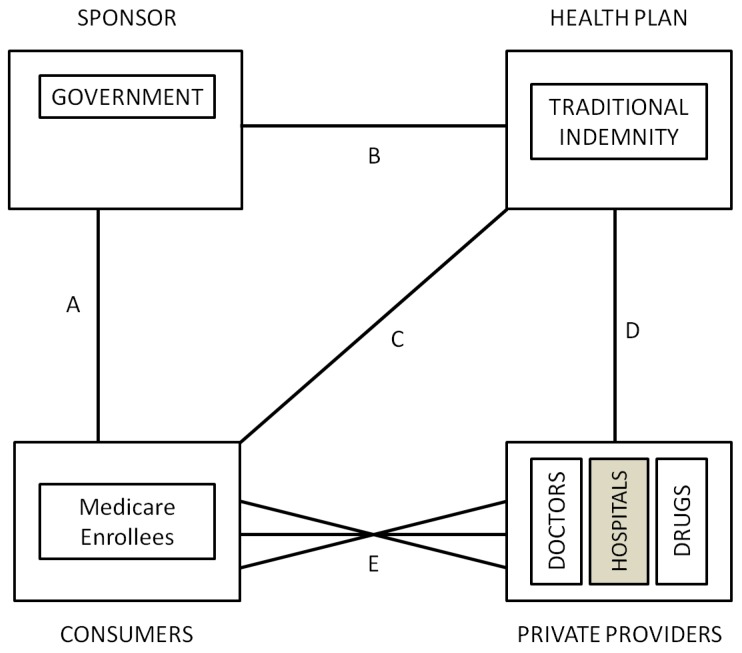
US medicare 1985.

The US Medicare program, which is available to every citizen and permanent legal resident who is either over age 65 or has a specified serious disability was introduced in 1965. Prior to 1985, when “at risk” HMOs were first permitted, the traditional indemnity Medicare program (US Medicare program covers aged, disabled or had end-stage renal disease people. As of December 2009, it covered nearly 47 million individuals.) was similar to the Canadian system, with a government sponsor raising revenue from taxes and insurance premiums, and fully insuring geographically-defined insurance carriers (health plans) that were contracted to pay services mostly on a fee-for-service basis (See Figure 3). The only real selection problem within the system was created by the introduction of DRG payments in 1983, which created incentives for hospitals to compete to avoid high cost/less profitable patients to the extent that they were able to. We are not aware of any evidence that selection into Medicare or between physicians in 1985 was viewed as a problem.

**Figure 4:**
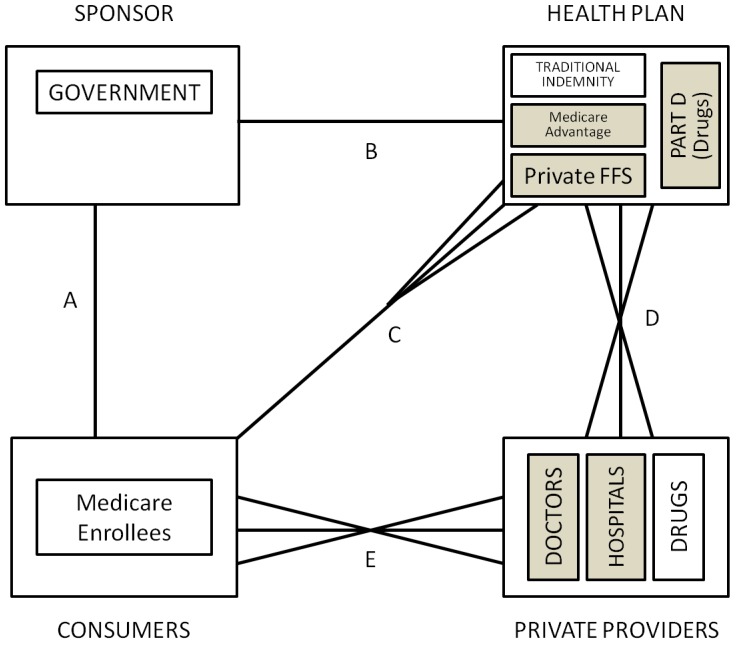
US medicare.

Changes adopted in 1985 to encourage cost containment by encouraging competing managed care health plans (now called the Medicare Advantage MA or Part C program. As of December 2009, MA programs covered almost 10.9 million people (23% of Medicare population).) permitted new types of Medicare health plans, namely HMOs and PPOs to receive capitation funding and be “at risk” for the cost of their enrollees. As shown in Figure 4, this program substantially expanded consumer choice, giving them the right to opt out of the traditional public Medicare Indemnity and to choose health plans directly. Health plans participating in this program are closely regulated in terms of the benefits they can offer and premiums they can charge. Open enrollment is required, but MA plans are allowed to compete in many other ways, including price (premiums), provider networks, geographic location and additional benefits (such as drug coverage). Originally, payments to the MA plans by the government were risk adjusted to reflect the county, age, gender, disability, and institutional status of the health plan’s enrollees, using a formula called the Adjusted Average Per Capita Cost (AAPCC). In 2000 the model transitioned to health status based risk adjustment using only Principal Inpatient Diagnoses called the PIP-DCG model [43]. In March, 2002, CMS selected an all encounter diagnosis model which they call the CMS-HCC model, a customized version of the Diagnostic Cost Group/Hierarchical Condition Categories (DCG/HCC) as described in Pope *et al.* [44], which is in turn based on classification system and organizational framework described in Ellis *et al.* [45] and Ash *et al.* [46]. The system is constantly being upgraded and new improvements were expected for 2012 (The CMS-HCC model’s explanatory power has been increased to 12.5% for the latest version to be implemented for PACE starting in 2012 [47].).

Although risk adjustment was intended to level the playing field and reduce incentives for health plans to focus on selection effort, in an effort to promote competition and foster increased choice in counties where the Medicare Advantage payments were low, administratively set prices were increased in more than 30% of US counties. Since prices no longer reflected expected costs, this fostered substantial new entry of health plans and a return to emphasis on selection as a profitable strategy.

In 2003 Medicare was expanded to include private prescription drug plans through a program called Medicare Part D. Key features of the Medicare Part D program include a late-enrollment penalty, a coverage gap known as the “donut hole” (The 2010 reform eliminates the Medicare prescription drug “donut hole” by 2020.), government subsidies including catastrophic coverage when expenditures are above a cap (2009’s cap was set at US $4,050) and risk adjustment. Some plans also offer layered coverage for different prescription drugs.

#### 5.2.2. Selection Problems in US Medicare

Since their inception, policymakers were concerned about whether risk adjustment using the “AAPCC” (which used only demographic information) was sufficient to reduce selection incentives and avoid overpayment of managed care plans [48,49]. Early evidence clearly indicated that the HMOs were attracting healthier than average enrollees even within each rate cell. The extremely important study by Brown *et al.* [50] concluded that rather than saving money, as intended, the Medicare managed care program was actually costing the Medicare program 5.7% more than it would have been if Medicare offered FFS alone without evident quality differences (It also showed that HMOs were able to significantly reduce the use of certain resources, such as inpatient days.). Moreover, Baker [51] highlighted not only were payments too high because of favorable selection by HMOs, but also because the FFS costs were biased upward. Greenwald *et al.* [52] and Cao [53,54] provide evidence of favorable selection in this program. More recent analysis has focused mostly to prescription drugs related selection. Heiss *et al.* [55] and Pizer *et al.* [56] raise concerns about the scope for risk selection within Medicare Part D, mostly through the choice of coverage level.

However, the major concern in the US about managed care plans is that selective contracting permits plans to distort services and provider availability in ways that encourage favorable selection [22,24]. Empirical evidence on the nature of this selection is limited. Cao [53] and Cao and McGuire [57] use Medicare FFS claims to detect that rates of spending on certain chronic diseases and certain services are higher in the FFS sector when a higher proportion of individuals are enrolled in HMOs, suggesting service and provider type distortions as predicted by the theory. Evidence of a different selection activity is provided in Dallek *et al.* [58] who find that Medicare managed care plans had primary care physician turnover rates averaging 14 %, with rates over 20% in five states. These extremely high turnover rates on primary care physicians must disrupt continuity of care, discouraging continued enrollment by those who are more seriously ill. In addition to concerns about selection induced service distortion, many policy makers are also troubled by the group access problem with Medicare, caused by the fact that not all counties have access to the same types of health plan choices. A minority of US policy makers and economists are concerned about the lack of income solidarity in the US, whereby lower income consumers have worse access to Medicare coverage because they are less likely to have a former employer sponsor who helps pay either their premiums or the cost of supplementary insurance (*i.e*., MEDIGAP) coverage.

#### 5.2.3. Role of Risk Adjustment and Risk Sharing and Plans for the Future

The US Medicare program reacted to the evidence of biased selection non-indemnity plans by promoting risk-adjustment in the early 2000’s. Pope *et al.* [43] describe the process and how several problems are addressed, including “up-coding” and reporting distortions. One particular line of concern in today’s model includes how much risk sharing would be optimal for the elderly to promote solidarity without affecting usage. Chandra *et al.* [59] suggest that health insurance should be tied to underlying health status, with chronically ill patients facing lower cost-sharing. Another line of concerns include payment for Medicare Advantage and how to reach financial neutrality (Berenson [60] highlights how difficult reaching financial neutrality can be at the local level with traditional Medicare).

Regulation regarding Part D is also a relatively new issue within the Medicare system. Since Part D plans are risk adjusted, one concern is the effect of this type of coverage on costs and usage given its strong incentive for adverse selection.

### 5.3. US Privately Insured

#### 5.3.1. Health Care System Overview

**Figure 5:**
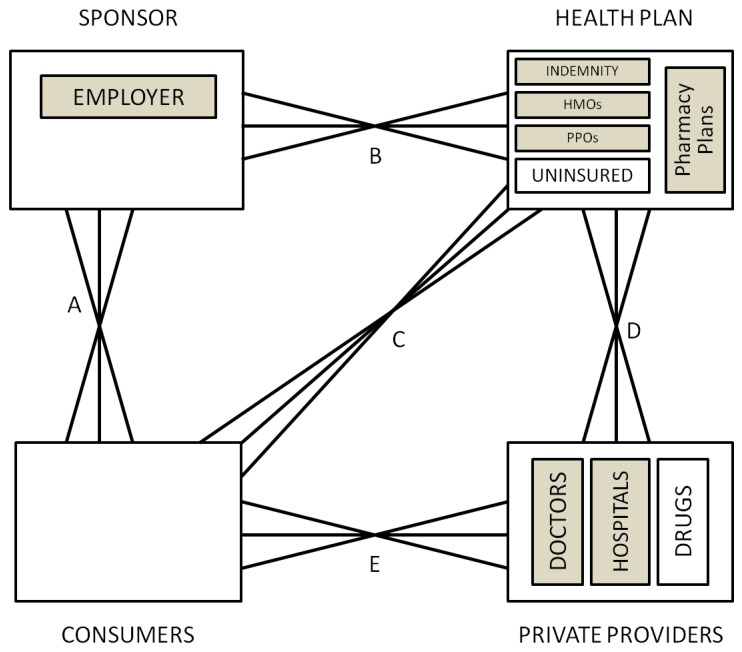
US private.

The US privately insured population has an extremely complex set of institutions providing health care. No simple overview can possibly capture its full complexity, although Cutler and Zeckhauser [61] provide a nice summary. For this paper, the most important insight about the US system is that it is a voluntary insurance program with elements of choice at every level. Even under the 2010 Affordable Care Act, which will require everyone to purchase health insurance in 2014 or else pay a tax, some people will voluntarily decline to purchase insurance. Figure 5 illustrates that each of the five contracting relationships is associated with elements of choice. The primary sponsor in almost all cases is the employer. Employers get to choose whether to offer insurance or not, and until the 2010 US health reforms there was no payroll tax or other penalty to employers who chose not to offer insurance in most states (The 2010 reform states that employers with more than 50 employees must provide health insurance or pay a fine.). Most employers subsidize the health plan premiums of their employees, if insurance is offered, although there are diverse ways that employers share the premium with their employees (linkage A). Employers are free to choose premium cost sharing levels separately for families and individuals and do so regularly (Consumers can purchase health insurance through their employer as a family, although most employers permit employees to choose not to insure spouses or other dependents [5].). Some employers offer (taxable) incentive payments for employees not to purchase insurance, often conditional on the employee showing that they receive coverage elsewhere (e.g., through a spouse’s employer-sponsored plan). All these characteristics influence employment choices.

Compared to most countries, the US relies upon linkage B, employers’ choice of health plans to offer to their employees, to control costs. Large employers typically offer one indemnity plans with broad coverage and a modest number of managed care plans, originally called HMOs. Because the utilization controls of HMOs were found to be unpopular in the 80s’ and 90s’ when HMOs were sometimes the only plan offered, a majority of workers are now offered more choice among two or more plans, with a diverse array of structures that rely on management, benefit plan design, selective contracting, and negotiated provider payment discount to varying degrees. Self-employed workers are able to buy individual policies, but in many cases choose not to, contributing to the uninsurance problem.

Since employees are often able to choose among multiple health plans, linkage C between health plans and consumers is also relevant in the US. Health plans advertise heavily on TV and in the media, and also through employers. Advertisements are common for HMOs, who generally are capitated and hence have strong selection incentives, showing the more typical advertisements of families with young children or athletes working out and other low cost individuals.

Health plans in the US have many tools available to affect selection. Most importantly, they may selectively contract with certain providers, and offer ex post incentive payments to providers according to the treatment patterns that occur. Health plans are free to choose provider payment mechanisms, and many formulas are in use. Plans are allowed to use utilization review or other management approaches that both affect costs and potentially act as selection tools. Plans can design benefits levels so as to discourage the chronically ill by not covering necessary testing supplies or durable medical equipment. Doctors and hospitals have considerable discretion in how they allocate their time among patients or accept new patients. Balance billing is allowed, but not common among the privately insured. Waiting times are short by international standards, although managed care plans have longer waiting times than indemnity, consistent with its use as a selection tool.

#### 5.3.2. Selection Problems

There is considerable evidence that selection problems abound in the US. First and foremost, in 2009 fully 18% of the US was officially uninsured, and hence is at financial risk for some or most of their health care spending. This is appropriately viewed as a selection problem in that surveys reveal that most of these uninsured are working, but relatively poor, and would prefer to have insurance: they just do not feel that it is worth the relatively high premiums (cost sharing with employers) that they must pay to become insured. Many of them use the “safety nets” of emergency rooms and state-funded “uncompensated care pools” to take care of urgent medical problems. This problem has been addressed by the 2010 Patient Protection and Affordable Care Act (often abbreviated simply ACA), which sets a fine for people that remain uninsured starting in 2014.

Adverse selection problems are also common among the privately insured. Cutler and Zeckhauser [61] summarize the evidence of biased selection among indemnity insurance plans while Glied [62] summarizes the evidence on selection problems with managed care plans. Miller and Luft [63] and Cutler and Reber [29] remain two of the most compelling studies in this area, with the later documenting a death spiral that occurs in the absence of a sponsor willing to sufficiently subsidize high risk enrollees. Ellis *et al.* [64] analyze claims from commercially insured and find evidence of service level distortions by HMOs to attract the healthy.

#### 5.3.3. Role of Risk Adjustment and Risk Sharing and Plans for the Future

The US is different from Canada and most European countries in that there is much less emphasis on solidarity and efforts to equalize access to health care. Health care is not uniformly viewed as a merit good to which all are entitled with the same level of access. Instead, permitting freedom of choice, and honoring individual heterogeneity of tastes has been revealed as a highly valued characteristic. Several provisions for directly addressing selection incentives present in the ACA of 2010—such as the introduction of fines for the uninsured, exclusions based on pre-existing conditions and the implementation of health insurance exchanges to facilitate access—are likely to change this scenario.

Although formal risk adjustment of payments between the sponsor and health plans is used for about 99% of all individuals insured by public programs in the US (Medicare, state Medicaid programs for the poor and medically needy, and state and federal government workers), it is used very infrequently by private employers [65]. The reasons for this are complex. A good summary of the factor explaining this complexity can be found in Glazer and McGuire [22], while Ellis [66] provides a discussion about the serious problems facing the introduction of risk adjustment for privately insured groups in the US and how this historic pattern could potentially change.

### 5.4. Colombia

#### 5.4.1. Health Care System Overview

**Figure 6:**
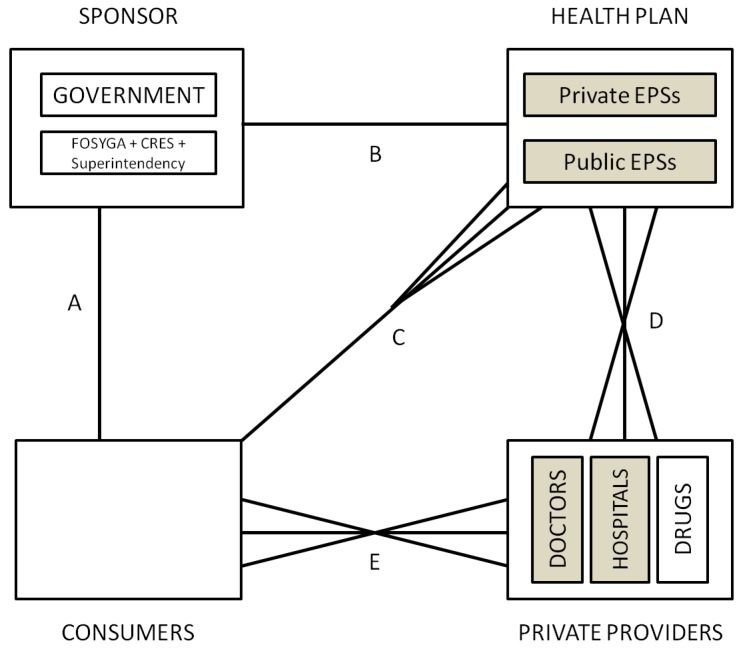
Colombia.

The basics of the Colombian system are depicted in Figure 6. The sponsor is an independent agency (CRES—*Comision de Regulacion en Salud*) (In 2007, the CRES was created to perform regulation functions previously under CNSSS (consejo nacional de seguridad social en salud) and included a technical department and full time experts. Although independent, members are designated by the president. It sets the capitated payment insurance companies receive for each affiliate, regardless of their usage and also regulates provider’s fees.). It sets the mandatory benefits of two standardized types of health plans: the regular mandatory health plan POS (*Plan Obligatorio de Salud*) and the subsidized health plan POSS (*Plan Obligatorio de Salud Subsidiado*) with lower coverage. Health plans are offered by both public and private insurance companies called EPS (*Empresas Promotoras de Salud*) and there is a national equalization fund called FOSYGA (*Fondo de Solidaridad y Garantia*) which transfers funds to EPSs based on a capitated adjusted payment formula (UPC -*Unidad de Pago por Capitacion*).

EPSs are in charge of the affiliation process, including the registration and the collection of contributions, which is then submitted to the FOSYGA. EPSs hire health care services with providers on behalf of the enrolled population (selective contracting is allowed). Providers can be public or private and are allowed to charge additional fees and copayments, except for the subsidized regime. Consumers are free to choose any provider in the EPS network. Finally, work related health care is financed by the employer through independent insurance companies called ARP (*Aseguradoras de Riesgo Profesional*) that rely on each employee EPS for providing care. ARPs must include sick leave among the benefits.

#### 5.4.2. Selection Problems

Despite the incentives present in the Colombian system, Alvarez [67] using data from 1997 finds no evidence of risk selection among EPSs. Selection problems have been mostly related to uninsured people, who still rely on the public network of providers. Despite being mandatory, informal and independent workers have the incentives to free ride and enroll only once they become ill. As regulation and enforcement of the Law is becoming tighter, these incentives are being lowered and the gap closed, with uninsured population decreasing from almost 40% to only 15% in 2007. Job market related inefficiencies are not a problem given the portability of health insurance, except perhaps for some minimal incentives to remain unemployed given the mean tested design of the subsidy for low income people [68].

Solidarity is one of the pillars of the Colombian system. The modest differential between coverage for contributors and for those under the subsidized regime limits the incentives for free riders to rely on the subsidized system. Moreover, the targeting of the subsidy has been one of the most efficient among social expenditures in Colombia, increasing medical care utilization among the country’s poor and uninsured [69,70].

Given the nature of the system, most insurance companies contract with providers in a managed care setting. Depending on the payment mechanism chosen by insurance companies, both public and private providers are likely to have incentives to select patients.

#### 5.4.3. Role of Risk Adjustment and Risk Sharing and Plans for the Future

Although the system presents a healthy level of improvement when compared to the system in place before the reform, still there is room for improvements [71]. One of the main tasks is to enroll the 15% uninsured population to improve risk sharing. Other permanent concern relates to financing and political pressure to improve the standardized plan beyond the ability to finance those improvements.

The main role for risk adjustment within the Colombian setting relies on the equalization fund and how it transfers money to each insurance company. Another possible role for risk adjustment is on payment mechanisms designed to transfer funds to providers. Finally, selective contracting and geographic variables remain as primary selection tools and should be studied.

### 5.5. Chile

#### 5.5.1. Health Care System Overview

The Chilean health system is basically organized as a mixed system with both private and public insurers and providers (for an historical perspective see Hudson [72]). Since 1981, the Law mandates that each worker allocates at least 7% of his income or pension to health insurance. Consumers usually purchase insurance as a family and must choose between a unique public insurance plan (FONASA—*Fondo Nacional de Salud*) and several insurance plans offered by private companies called ISAPREs (*Instituciones de Salud Previsional*). Low income people (non-contributors) are covered by the public plan by default, which is financed mostly through direct transfers from the general government (58.4% of FONASA’s income in 2011) and the 7% contributions of its enrollees (As of December 2011, 76.2% of the population was covered by FONASA, while 16.9% was covered by the private ISAPRE subsystem after a peak of 26% in 1997.).

In 2005 the system was reformed in several ways, the most important being the prioritization of selected health conditions referred to as AUGE conditions (*Acceso Universal de Garantias Explicitas* or Explicit Guarantees and Universal Access) (The priority refers to financial coverage, timely treatment and quality of care). The public and private subsystems must offer coverage for treating the same AUGE conditions as a special item (Usually treated only through preferred providers under selective contracting in the private system. Publicly covered patients that do not receive timely treatment can be transferred to private institutions). The AUGE related premium is common to all plans offered by each insurance company and there is an Inter-ISAPRE compensatory fund for AUGE related conditions, risk adjusted based on age and sex.

**Public Subsystem**: The public sector acts not only as the last resort and main insurer, but also as the largest health care provider in the country, integrating the sponsor, the health plan and the provider roles. Institutional related services are provided by a network of public hospitals and primary care clinics, with different levels of complexity, organized under the National System of Health Services (SNSS “Servicio Nacional de Salud”). Primary care services are usually independent from the SNSS and relatively well organized, delivering medical, dental, nursing and midwifery services at local health centers administered and owned by local governments called municipalities [73,74]. Financing for institutional primary care comes from FONASA through the regional services to the municipalities with approximately 60% of it being adjusted capitated transfers (adjusted based on population’s age, sex, socioeconomic conditions and whether it is a rural or urban area). Institutional secondary and tertiary care is provided by hospitals and clinics that depend on the SNSS through regional health services directly. Institutional providers under the public system offer either free or low cost services depending on the socioeconomic characteristics of the patient, but usually with long waiting lists and lower quality, especially for non-prioritized conditions.

**Figure 7:**
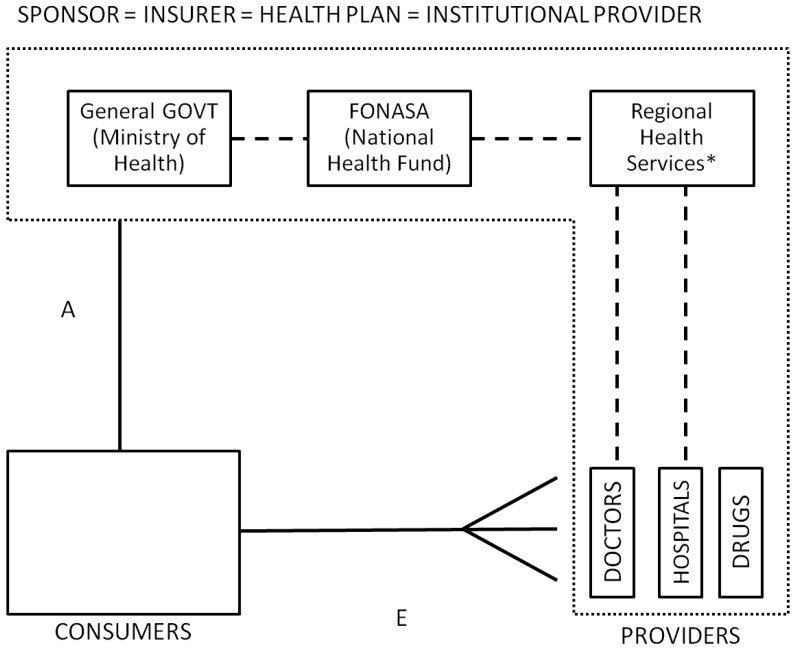
Low-income fully subsidized public system, Chile.

**Figure 8:**
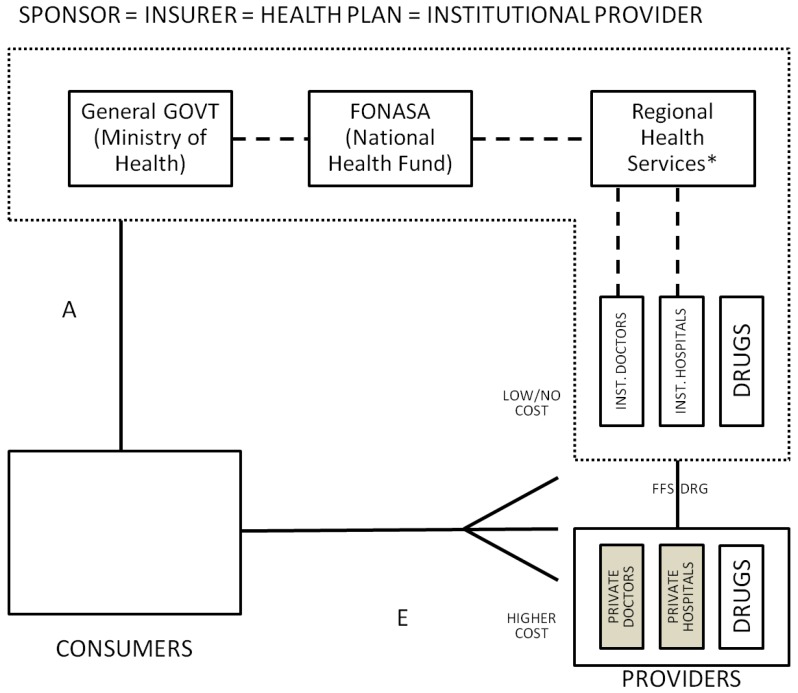
Public system, Chile.

FONASA non-contributors (also called indigents) must use the institutional/public network of providers (Figure 7), while contributors and their dependents can opt to visit affiliated private providers through a “free-choice” alternative at a regulated fee (see Figure 8). Private providers must sign an agreement with FONASA to become affiliated “free-choice” providers (linkage D), stating which and how many services will be provided. FONASA publishes each year a table with the fees providers can charge. “Free-choice” related services are paid either on a fee-for-service scheme or as a bundle for selected DRGs (PAD or “*Pago Asociado a Diagnostico*” program). While the public health care provider network spans all regions of Chile, private health care providers are concentrated primarily in urban areas.

**Figure 9:**
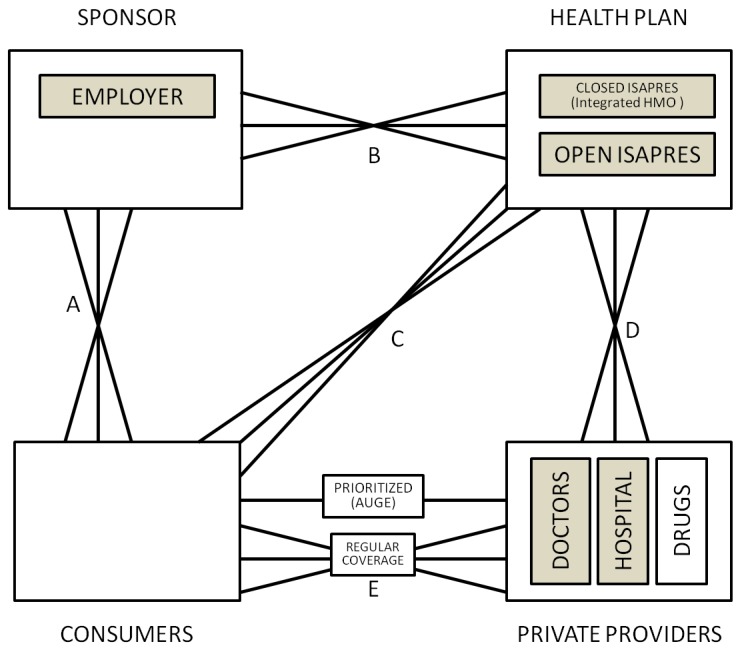
Private system, Chile.

**Private Subsystem**: Within the private subsystem (Figure 9), the insurance companies (ISAPREs) offer several plans to consumers and if the cost of the desired plan is above the mandatory 7% contribution, consumers can agree on a supplemental payment (This additional contributions financed 27.94% of the ISAPRE subsystem in 2011.). Plans are characterized by a “table of adjustment factors” and coverage for both outpatient and inpatient services (which can differ if a preferred provider exists, but cannot be lower than the benefits offered by FONASA). The non-AUGE part of the cost of each plan is known as additional coverage and is calculated per capita based on a unique base-payment (function of each plan’s coverage), times a factor function of age, sex and whether the individual is a dependant or a contributor. Each ISAPRE can have only two tables for all the plans being offered [75].

Plans are allowed to reject patients based on preexisting conditions. ISAPREs use a health status questionnaire, used to reject patients or to explicitly lower coverage for specific conditions. Once enrolled, ISAPREs are not allowed to drop patients. The public system acts as the insurer of last resort for those who can’t afford private insurance, are not accepted or consider it too expensive.

Private providers can sign an agreement with the ISAPREs to be part of their network or to become preferred providers. In general, there is no regulation as to how much a doctor can charge when treating privately insured patients and they are usually paid on a fee for service scheme. For more complex treatments and AUGE conditions there is usually a bundled payment, which can be complemented with fee for service for additional procedures.

Although there are several supplemental insurance plans offered by some private clinics and insurance companies that are not ISAPRE, the most common one is offered as part of the ISAPRE’s plans and is called CAEC (*Cobertura Adicional de Enfermedades Catastroficas*). It is a catastrophic disease insurance designed to alleviate the burden of high cost diseases after a deductible has been reached. It works only when patients are treated by preferred providers (managed care).

#### 5.5.2. Selection Problems

Sapelli and Vial [76] find evidence of adverse selection between the public and the private systems in the mid nineties. Based on the choices available to each of the four main agents there are many selection problems still present in the Chilean health system, most of which are between the private and the public subsystems at the patient level (limited by the high subsidies embedded in the public system). Private plans are allowed and have the incentives to reject expensive patients. In order to achieve selection among existing customers, plans can either modify their coverage, increase prices and/or offer new plans to affiliates who are willing to change (Discounts to a subset of clients of a plan was forbidden in the 2005 reform.). The higher priced private health care and the existence of caps and co-payments also discourage sick people. The existence of the FONASA alternative at a 7% premium also incentivizes large families to switch. Finally, despite efforts to standardize the supply of private health plans, the large number of plans and their complexity make comparisons hard to understand and allow for stinting. In this framework, the main mechanism used for selecting high income people is through linkage D and the selection of high quality providers.

On a related issue, there is an increasing concern about the priority given to certain conditions (AUGE) and how it affects selection. AUGE regulation can potentially generate inequality between affiliates based on their disease and age, generating also long waiting lists for non-prioritized conditions, mostly in the public subsystem.

Since funding for all public institutions is channeled through the general government (except for copayments and a portion of primary care funded by local governments), selection problems and solidarity concerns are limited within the public subsystem. FONASA has no room for selection when acting as a sponsor because coverage is the same for everyone and there is a unique plan, with only two delivery methods. Job market frictions are not present either: contributing 7% is mandatory and insurance is fully portable. Potential sources of selection are the contracting process between private providers and FONASA, patient referrals and patient geographic allocation plus the behavior of independent workers that use the public system as an insurer of last resort (Low fees attract mostly providers trying to build reputation or that are of low quality (plus dedicated doctors with a different primary source of income).). In this framework, the most relevant concerns are the design of disbursement and payment mechanisms to promote efficiency in public institutions and to provide a fair patient allocation with a reasonable geographic coverage.

#### 5.5.3. Role of Risk Adjustment and Risk Sharing and Plans for the Future

A relevant question in Chile is whether measures aimed at controlling selection should be implemented broadly between subsystems or within subsystems given its segmented nature and the high income inequality. Moreover, given the financing structure of the market it is hard to argue in favor of a simplistic view, mostly because there are hidden subsidies between subsystems through providers that are willing to work for a low wage in the public system as long as they keep their private practice. Wasem and Vargas [77] and Blackburn *et al.* [78] analyze how risk adjustment can be implemented in this environment and how to deal with health plans that consistently use providers that differ both on their quality level and payments, in the presence of segmentation even within the private system and large variation in plan features. A more long term concern given the incentives to risk select between systems is how to include the public sub-system in the risk adjustment process. Regardless of the solution, it will have to address the fact that while public providers are funded through general taxes, private ones are mostly financed by health plans and risk adjustment can be seen as a tax. Regarding risk sharing, the most relevant discussion within the private subsystem is whether the adjustment factors table should exist at all (currently under debate after a 2010 trial that ruled their current structure unconstitutional) and how to facilitate health plan comparisons.

### 5.6. America’s Experience: Synthesis

The figures and text in the preceding section have summarized the principal health care institutions in each country, by which we mean the principal decision making agents, the dimensions of choice in each country, and the major payment flows. We have also presented what we feel are the primary selection tools available to each agent, and the primary regulations and financial tools used to mitigate selection. The challenge for this section is to attempt to draw some useful conclusions out of all of this complexity.

In an attempt to further summarize the diverse experience of different countries and health care networks, in Table 1 not only do we present the selection problems described in the first section on this paper, but also information on each country in a simplified form. Table 2 presents the choices available for each agent, while Table 3 shows selection tools, or how the previous choices can be used to select patients. Each column in each table represents a different insurance network, in some cases a single system (Alberta, Canada or Colombia), in others a specific program (US Medicare 2003). At the risk of being provocative, we have ordered the countries/systems to have those with the less serious selection problems on the left and those having the most serious problems on the right. Canada and the public subsystem in Chile are not viewed as having any substantial selection problem, whereas the Chilean private subsystem and US privately insured consumers are classified as having problems with all four types of problems. Colombia is in between these two extremes.

The prevalence of X’s in each table gives an impression of how carefully the government or other regulators have attempted to minimize selection tools and problems. It seems that the larger the choice levels available in a system for different agents, the greater the selection concerns. Reviewing all of the different selection tools available would lead us astray, but it is interesting to note that even Canada and the US Medicare program in 1985 had to deal with geographic variation as “selection problems” due to non-random enrollee sorting. The picture highlights that the institutions in various countries are quite different, and hence the selection problems are as well. In the USA and Chile, selective provider contracting is pervasive, and hence there are enormous concerns about selection motivated service distortion. As countries attempt to create more incentives for cost containment, they need to be alert to whether they are also creating greater selection incentives. The central selection problems that countries worry about differ dramatically between the USA on the one hand and Canada on the other hand. In the USA, risk and income solidarity are not viewed as central issues thus far, and relatively large differences in premium contributions across consumers are tolerated. In contrast, in Alberta Canada, this solidarity is viewed as central. Chile and Colombia are in between.

## 6. Conclusions

The preceding sections have summarized the principal decision making agents, the dimensions of choice of each of these agents, and the type of selection concerns each of these choices can raise within health care systems. We have also presented the main choices through which selection is possible for each agent, and a sampling of the regulations and financial tools used to mitigate selection. Examples from Germany, Netherlands the US, and Canada are provided in Ellis [79], while this paper presents a discussion of applications in four countries of the Americas, including an overview of the choices allowed, the selection problems faced, and the regulations in place to reduce these problems.

We believe that we have made four contributions or innovations in this paper. The first innovation has been to emphasize the choices that are available to each agent, since these choices are the fundamental sources of selection problems. One important set of choices are the choices among agents, which on our figure is represented by multiple lines, facilitating a more graphic description of different health care systems. We have also identified the selection tools available to each agent that may possibly be used to influence these choices.

The second contribution of this paper has been to highlight the ways that regulations can be as important as risk adjustment and risk sharing methods in influencing risk selection. We show that regulatory policies both create and ameliorate risk selection, and in some cases are as important as risk adjustment and risk selection strategies for reducing risk selection. While we are firm believers in improved risk adjustment and risk sharing techniques, however at the same time it is important not only to worry about the financial incentives, but also about the setting in which these tools are implemented. This observation also justifies sensitivity in how these financial instruments should be implemented in different settings.

A third contribution has been to clarify the types of selection problems that are perceived to be of importance and how they can depend on the market structure. The examples we present can also be useful to understand how this framework can be useful for analyzing different health care systems. Canada, Chile, Colombia and the US differ not only in their health care institutions, but also in their objectives. It is a mistake to try to characterize how well each country is doing without acknowledging the differences in objectives. Building on the three selection concerns of Glazer and McGuire [24] (individual access, group access, and service distortion) we have added “risk and income solidarity” as an important objective relevant to selection concerns.

The fourth and final contribution of this paper has been to present a stylized description of the problems and challenges with respect to selection concerns present in four different health care systems: Canada, Chile, Colombia and the US. A reasonable criticism of this paper is that we emphasize selection problems and the tools available to deal with them, but we have not described the cost and quality incentives of using these tools, nor the efficiency effects. To a great extent, the same selection tools shown in Table 3 are also important as tools to promote efficiency and quality of care. It would have to be a separate paper to examine the trade-offs between these different objectives. We must assert, however, that we do not believe that the countries with the largest selection problems are those that have achieved the lowest costs or highest quality. The challenge for future policy makers, where cost containment and quality are clearly important objectives, is to figure out mechanisms for promoting these objectives without sacrificing access and solidarity objectives that can be compromised by selection.
